# Expression of Concern: Downregulation of microRNA-142-3p inhibits the aggressive phenotypes of rheumatoid arthritis fibroblast-like synoviocytes through inhibiting Nuclear factor-kappa B signaling

**DOI:** 10.1042/BSR-20190700_EOC

**Published:** 2020-06-25

**Keywords:** MicroRNA-142-3p, Inflammation, Proliferation, Tumor necrosis factor α, Nuclear factor-kappa B

The Editorial Office has been made aware of potential issues surrounding the scientific validity of this paper, hence has issued an expression of concern to notify readers whilst the Editorial Office investigates.

